# Editor’s Note: Hyaluronan Is Crucial for Stem Cell Differentiation into Smooth Muscle Lineage

**DOI:** 10.1093/stmcls/sxag055

**Published:** 2026-10-13

This is an Editor’s Note regarding: Russell M.L. Simpson, Xuechong Hong, Mei Mei Wong, Eirini Karamariti, Shirin Issa Bhaloo, Derek Warren, Wei Kong, Yanhua Hu, Qingbo Xu, Hyaluronan Is Crucial for Stem Cell Differentiation into Smooth Muscle Lineage, Stem Cells, Volume 34, Issue 5, May 2016, Pages 1225–1238, https://doi.org/10.1002/stem.2328

In September 2026, the journal published an Expression of Concern^1^ regarding apparent similarities between Figures 3G and 4F. The journal investigated these concerns in line with guidance from the Committee on Publication Ethics (COPE), including contacting the corresponding author’s institution at the time of article publication, which confirmed that the data have since been destroyed. While the issues described above may not affect the results or conclusion of the study, in the absence of the original data, the editor advises readers to examine these figures with care.

## Footnotes

1 Expression of Concern: Hyaluronan Is Crucial for Stem Cell Differentiation into Smooth Muscle Lineage, Stem Cells, Volume 44, Issue 10, October 2026, sxag050, https://doi.org/10.1093/stmcls/sxag050

